# Multiphoton tomographic analysis of hyaluronic acid delivery: comparison of carbon dioxide laser and 1927 nm thulium laser over time

**DOI:** 10.1007/s10103-025-04363-5

**Published:** 2025-02-17

**Authors:** Lynhda Nguyen, Christian Mess, Stefan W. Schneider, Volker Huck, Katharina Herberger

**Affiliations:** 1 Laser Department, Department of Dermatology and Venereology, University Medical Center Hamburg-Eppendorf, Martinistrasse 52, 20246 Hamburg, Germany; 2 Department of Dermatology and Venereology, University Medical Center Hamburg-Eppendorf, Martinistrasse 52, 20246 Hamburg, Germany

**Keywords:** Laser-assisted drug delivery, CO_2_ laser, Thulium laser, Hyaluronic acid, Non-invasive imaging

## Abstract

Hyaluronic acid injections, known as skin quality boosters (SQB), improve skin hydration and quality. Laser-assisted delivery using ablative (AFL) and non-ablative fractional lasers (NAFL) enhances the uptake of topical drugs and cosmeceuticals. This study aims to compare the biodistribution of SQB as well as the morphologic and metabolic cellular changes following CO_2_ and thulium (Tm) laser treatments. Healthy male and female volunteers were assigned to either the CO_2_ laser or the Tm laser treatment group. Areas on their forearm were marked for following treatments: laser with SQB application, laser monotherapy, topical SQB monotherapy, and untreated control. Measurements were carried out 30 min and 30 days post-treatment using multiphoton-tomography equipped with fluorescence lifetime imaging (MPT-FLIM). Eleven volunteers were included in each group. SQB penetration reached the upper dermal layer in both groups, with larger aggregates after CO_2_ treatment. The CO_2_ laser induced a higher level of inflammation, which persisted as subclinical inflammation even 30 days post-treatment. In contrast, skin treated with the Tm laser showed a metabolic state similar to the untreated control after 30 days. Both CO_2_ laser and Tm laser enhance cutaneous uptake of the SQB, potentially improving skin hydration and quality. While the CO_2_ laser may be more effective in delivering SQB, the Tm laser may be a treatment option with minimal downtime. MPT-FLIM proved to be a suitable tool for monitoring treatment outcomes.

## Introduction

Hyaluronic acid (HA) is extensively used in dermatology for enhancing skin quality. In addition to HA-containing topicals, lightly cross-linked HA injectables, known as skin quality boosters (SQBs), are also utilized. These SQBs are injected into the dermal and subdermal layers of the skin to provide hydration and improve the appearance of photoaged skin. However, a major drawback of this treatment is the presence of bumps for several days after the procedure [1].

Laser-assisted drug delivery (LADD) is a highly effective technique to increase dermal uptake of topically applied pharmaceuticals and cosmeceuticals. Since its first introduction in 2009, its application expanded in the field of both dermatology and pharmacology [2]. Ablative fractional lasers (AFLs), like the carbon dioxide (CO_2_) laser, have been the most applied systems for delivering topicals to and through the skin [3]. By creating microscopic treatment zones in a controlled manner, these microchannels provide an increased permeability and penetration of various applied topicals. A previous study has documented the microscopic analysis of increased biodistribution of SQBs after ablative fractional CO_2_ laser [4].

In recent years, there has been growing interest in more minimal to non-invasive treatment options [5]. Unlike AFL treatments, non-ablative fractional laser (NAFL) procedures, like the Thulium (Tm) and Erbium-Glass laser, do not or minimally disrupt the stratum corneum, resulting in less downtime for patients [6]. However, limited data are available on NAFL-assisted delivery of SQBs at a microscopic level. High-resolution, non-invasive, intravital imaging techniques like multiphoton tomography equipped with fluorescence lifetime imaging (MPT-FLIM) are necessary to adequately evaluate microscopic morphologic alterations and biodistribution of topicals [4, 7]. Based on two-photon autofluorescence and second harmonic generation (SHG), this imaging system enables visualization of skin and drug delivery, as well as altered metabolism of the surrounding tissue [8–10].

Currently, our knowledge about SQB uptake in living human skin is limited to clinical evaluations only [11]. Therefore, our study aimed to evaluate the biodistribution as well as morphologic and metabolic alterations in in vivo human skin following CO_2_ and Tm laser-assisted delivery of HA using MPT-FLIM.

## Material and methods

### Study design

This study was designed as a comparative, prospective study. It was approved by the local ethics committee (2022–100893-BO-ff; 2023–101110-BO-ff), preregistered in clinicaltrials.gov (NCT06431282), and applied in accordance with the Good Clinical Practice and Declaration of Helsinki. Healthy female and male subjects were recruited. Exclusion criteria were pregnancy, breast feeding, open wounds or lesions at the area to be treated. After informed consent, subjects were assigned to a laser treatment using a fractional 10,600 nm CO_2_ laser or a fractional 1927 nm Tm laser with and without subsequent application of a weakly-crossed HA preparation. Subjects were measured 30 min (visit V1) and followed-up 30 days (visit V2) after treatment using an MPT-FLIM system. Data on the CO_2_ laser treatment group were published previously [4].

### Treatment protocol

For each volunteer, four squares were marked on the right or left forearm: (i) laser with subsequent SQB application, (ii) laser monotherapy, (iii) SQB application monotherapy, and (iv) untreated control. The squares were marked on a transparent foil to ensure consistency in follow-up measurements.

Prior to the CO_2_ laser treatment (eCO2 Plus™, Lutronic Medical Systems, Hamburg, Germany), participants received a local anesthetic ointment (23% lidocaine, 3.5% tetracaine, 3.5% tetracaine-HCl) under occlusion on the area to be treated for 30 min. After thorough cleansing and disinfection, participants were treated with the following parameters: 120 µm, 40 mJ, 30 W, 8 mm square, 5.8% density. For the Tm laser treatment (LaseMD Ultra^TM^, Lutronic Medical Systems, Hamburg, Germany), following parameters were applied: C1 handpiece, random mode, 5 mJ, 6 passes. Here, local anesthesia was not necessary.

Directly after laser treatment, 0.5 ml of an SQB (Restylane Vital light® Skinboosters™, Galderma®, Lausanne, Switzerland) was applied on the marked squares under occlusion for 30 min. Participants were advised to avoid extensive UV exposure or HA-containing topicals on the treated areas for 30 days post-treatment.

### Multiphoton tomography with fluorescence lifetime imaging

A CE-certified MPT-FLIM system (MPTflex, JenLab GmbH, Berlin, Germany) was applied. We used a titanium:sapphire tunable laser system (Mai Tai, Newport Spectra-Physics, Santa Clara, CA, USA) to provide near-infrared laser pulses for excitation of endogenous fluorophores and inducing SHG. The technical setup consisted of a Glan calcite polarizer, two galvanometric mirrors, a beam expander, and a collimator. Laser pulses were reflected into a 40 × oil immersion objective with a numerical aperture of 1.3 (Carl Zeiss Jena GmbH, Jena, Germany). Photomultipliers (PMT) combined with time-correlated single-photon counting (TCSPC) were used for image acquisition.

### Data acquisition

Biogenic fluorophores and SQB were excited at a wavelength of 760 nm. For each region of interest, two or three z-stacks of 100 µm × 100 µm images were acquired from the corneal to the dermal layer up to a depth of around 150 µm with 1.5 µm increments. We performed time-correlated single photon counting (TCSPC) using a TCSPC module (SPC 830, Becker & Hickl GmbH, Berlin, Germany) and analyzed the resulting data using SPCImage 8 software (SPCM, Becker&Hickl GmbH, Berlin, Germany). Mean fluorescence lifetimes (τ_m_) were calculated to objectively assess the inflammatory cellular state and was defined as [10, 12]:

$$\tau m=\frac{a1\tau 1 + a2\tau 2}{a1 + a2}$$

τ_1_ represents the lifetime of free NADH, while τ_2_ corresponds to the lifetime of protein-bound NADH, with a_1_ and a_2_ denoting their ratios. The data was then pseudo-colored (from 100 ps (red) to 2,000 ps (blue) for illustrative purposes.

### Statistical analysis

We performed the statistical analysis using GraphPad Prism (Version 9, Graphpad Software, Boston, USA). Descriptive data are presented as means ± standard deviation and ranges (minimum – maximum). The paired t-test was used to determine the difference in mean between groups unless stated otherwise. *P*-values < 0.05 were considered significant.

## Results

### Baseline characteristics

Eleven volunteers were included in each group, though one volunteer in the Tm laser group was lost to follow-up. The average age was 25.1 ± 7.3 years in the CO_2_ laser group and 31.8 ± 9.2 years in the Tm laser group.

### Histomorphological evaluation

**Fig. 1:**
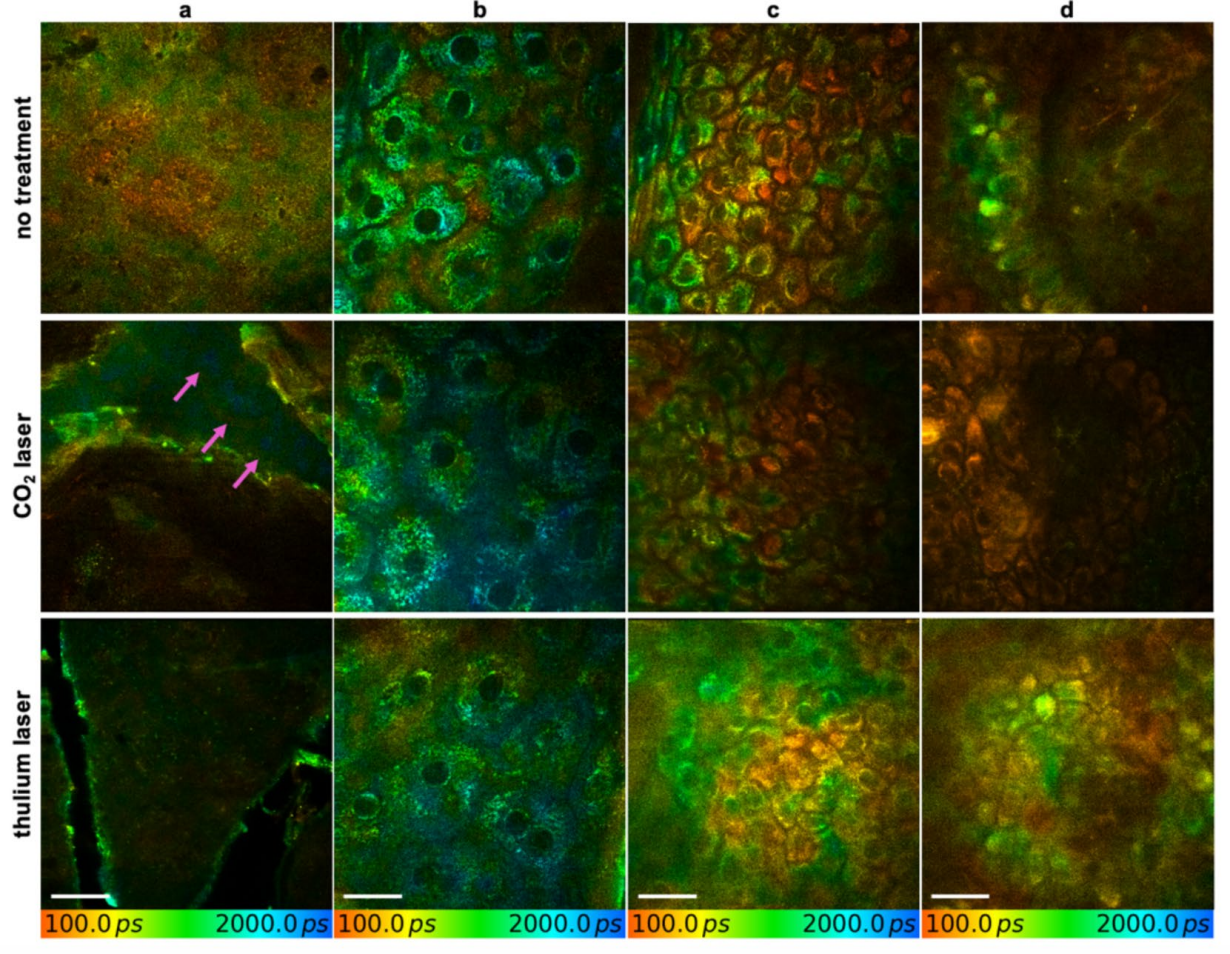
Multiphoton fluorescence lifetime imaging of untreated skin and skin treated with CO_2_ laser- and thulium laser 30 min post-treatment. Figure depicts (**a**) the corneum to upper granular layer, (**b)** the granular layer, (**c)** the spinal layer, and (**d**) the dermal layer. Altered morphology, like irregular intercellular spaces and swollen keratinocytes, suggests inflammation following laser treatment. Pink arrows indicate bluish fluorescence signal, marking presence of anesthetic ointment. Scale bar: 20 µm

30 min after CO_2_ and Tm laser treatment, characteristic morphologic alterations of inflamed skin were observed. The irregularly shaped keratinocytes and broadened, intercellular spaces clearly indicate laser-induced edema (Fig. 1). The edema was more pronounced after CO_2_ laser treatment compared to Tm laser treatment and caused laser scattering of the MPT, limiting deeper visualization of the skin during visit V1. After 30 days, the histomorphology of the epidermis and upper dermis in both laser groups was similar to that of healthy, untreated skin, with MPT images showing regular inter- and intracellular architecture.

Furthermore, a bluish fluorescence signal with a lifetime of approximately 2,600 ps was detected within wrinkles and intercellular spaces, indicating the presence of the anesthetic ointment applied prior to treatment. This signal was exclusively observed in the CO_2_ laser group, as the Tm group did not require anesthesia before treatment. Anesthetic ointment was no longer detectable at visit V2.

### Follow-up of penetration depth of hyaluronic acid

**Fig. 2:**
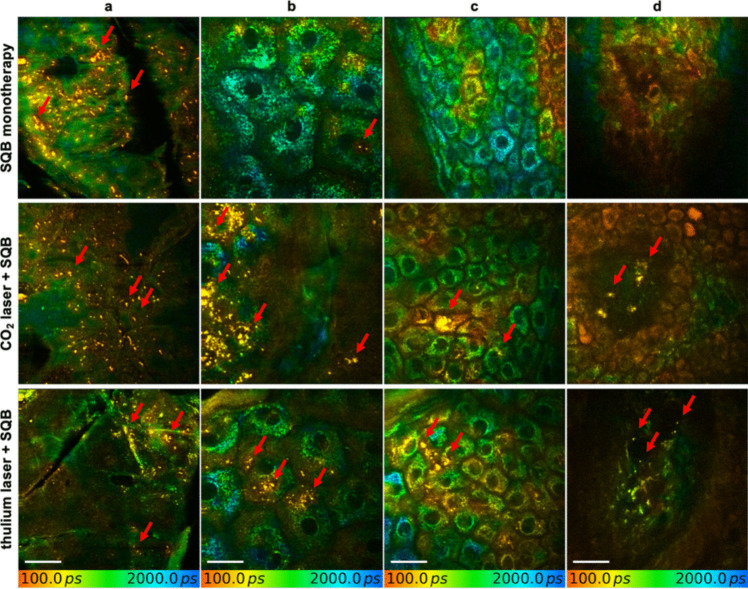
Visualization of hyaluronic acid (HA) distribution in in vivo human skin using multiphoton fluorescence lifetime imaging, following topical application only, CO_2_ laser treatment, and thulium (Tm) laser treatment. Images indicate (**a**) the corneal to upper granular layer, (**b)** the granular layer, (**c)** the spinal layer, and (**d**) the dermal layer. Topical application alone led to a penetration only to the upper granular layer. Laser pretreatment significantly enhanced cutaneous uptake, extending to the dermal layer. The size of HA aggregates was similar in skin without pretreatment and with Tm laser pretreatment. In contrast, following CO_2_ laser treatment, HA aggregates varied widely in size and were significantly larger compared to the other groups. Arrows indicate HA aggregates. Scale bar: 20 µm

**Fig. 3:**
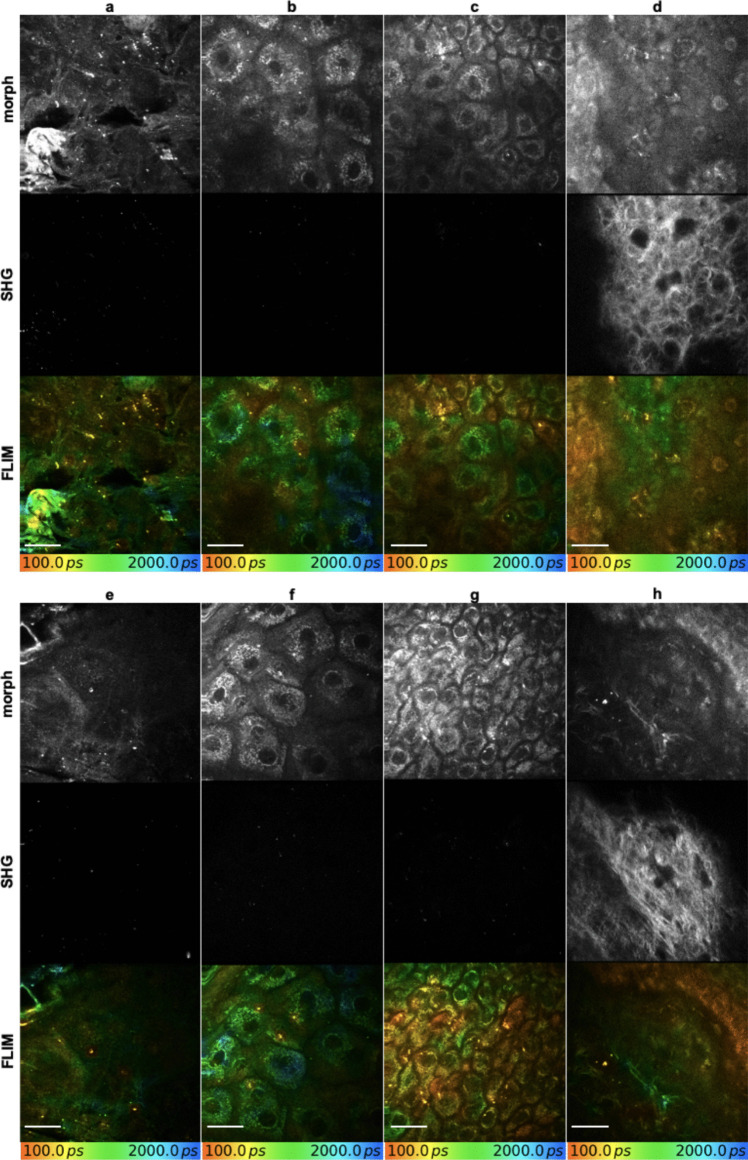
30 days after CO_2_ laser- (**a – d**) and thulium (Tm) laser-assisted delivery (**e – h**) of hyaluronic acid (HA) into human skin visualized using multiphoton fluorescence lifetime imaging (MPT-FLIM). HA remained detectable weeks after treatment in the (**a, e**) corneal layer, (**b, f)** granular layer, (**c, g)** spinal layer, and (**d, h**) dermal layer. Figure display morphological aspects in MPT-FLIM, second harmonic generation imaging of collagen bundles, and pseudo-color coded FLIM. Scale bar: 20 µm

MPT-FLIM allowed SQB to be distinguished from surrounding tissue with a mean fluorescence lifetime of 80 ps (Fig. 2), which was defined as τ_3_ in further calculations. Topical application of SQB alone resulted in limited penetration, reaching the upper granular layer at depths of 21.3 ± 5.0 µm (16.5 – 28.5 µm) in the CO_2_ laser group and 23.2 ± 6.5 µm (15.5 – 22 µm) in the Tm laser group (*p* = 0.586). Pretreatment with a fractional laser significantly enhanced product uptake. After CO_2_ treatment, SQB penetration increased markedly, reaching depths of 82.1 ± 7.1 µm (73.5 – 90 µm; *p* < 0.001) within collagen bundles. Similarly, in Tm-pretreated skin, SQB was visualized at depths of 81.6 ± 11.5 µm (63.0 – 90.0 µm; *p* < 0.001) within collagen bundles. There was no significant difference in penetration depth between the two laser systems (*p* = 0.909). At 30 days post-treatment, SQB was still detectable in both CO_2_- and Tm-treated skin across all epidermal layers and the upper dermis (Figs. 3).

### Dispersion of hyaluronic acid

The SQB granules size in untreated skin were similar in both laser groups, measuring 1.1 ± 0.3 µm (1.0 – 2.0 µm) in the CO_2_ laser group and 1.1 ± 0.4 µm (1.0 – 2.0 µm) in the Tm laser group. After CO_2_ treatment, SQB granules in the granular layer were 9.6 ± 13.3 µm (1.0 – 40.0 µm) in diameter, decreasing with depth to 4.3 ± 4.7 µm (1.0 – 15.0 µm) in the spinal layer and 1.8 ± 1.0 µm (1 – 4 µm) in the dermal layer. In contrast, after Tm treatment, SQB aggregates were significantly smaller: 2.0 ± 0.9 µm (1–4 µm) in the granular layer, 1.3 ± 0.5 µm (1–2 µm) in the spinal layer, and 1.3 ± 0.5 µm (1–2 µm) in the dermal layer. As observed initially, 30 days post-treatment, diameter of SQB aggregates remained larger in CO_2_-pretreated skin 1.5 ± 0.6 µm (1.0 – 3.0 µm) compared to Tm-pretreated skin 1.2 ± 0.4 µm (1.0 – 2.0 µm) in the dermal layer.

### Follow-up of the metabolic cellular state

We analyzed the metabolic cellular state of the granular layer by performing a three-component fit on the TCSPC data. This allowed us to catch the SQB signal with a very short lifetime of 80 ps. After removing the SQB lifetimes from the data set, we calculated a mean fluorescence lifetime τ_m_ out of the two remaining NADH components. Our observations revealed a clear increase in τ_m_ in both CO_2_- and Tm-exposed skin, with a significant rise by 130 ± 32 ps (114%) in CO_2_-exposed skin and 96 ± 17 ps (110%) in Tm-exposed skin compared to control at visit V1, indicating acute inflammation.

In skin treated with CO_2_ monotherapy, after 30 days, τ_m_ decreased by 40 ± 25 ps (110%) indicating a persistent but subacute inflammation. With subsequent SQB application, τ_m_ further showed a decrease of 90 ± 34 ps (103%). In comparison, in the Tm treatment group, τ_m_ decreased to the value of untreated control with reductions by 91 ± 28 ps (101%) without SQB and 93 ± 20 ps (100%) with SQB application. There was no significant difference between Tm monotherapy and additional SQB application, nor between Tm treatment with and without SQB application and untreated control.

## Discussion

The data showed that CO_2_ laser treatment resulted in significant cellular and metabolic alterations, as well as enhanced HA uptake, compared to Tm laser treatment over an extended period. This study is the first to provide an in vivo comparative analysis of laser-assisted delivery of HA in human skin following both laser treatments using multiphoton fluorescence lifetime images.

The cutaneous bioavailability of most pharmaceuticals and cosmeceuticals is limited. In fact, only 1 – 5% of these substances are absorbed into the skin [13]. Many agents, like HA, are too large to penetrate the skin and require pretreatment of the skin through injections or systematic delivery. Laser-assisted delivery is an evolving treatment modality to allow a greater penetration depth of topical medications [3].

In recent years, treatments to enhance skin quality gained in popularity. While the injection of HA can lead to multiple bumps and an associated downtime of several days, fractional lasers may offer a less invasive treatment option. As previously discussed, CO_2_ laser pretreatment significantly enhances the cutaneous uptake of HA compared to no pretreatment [4]. The data clearly demonstrate that Tm laser pretreatment using a 1927 nm Tm laser also increases penetration depth down to the upper dermal layer as evident from the MPT-FLIM images. We could not identify a significant difference in the depth of cutaneous uptake in both treatment groups. However, size of SQB aggregates differed distinctly. While after CO_2_ laser treatment, there was a wide variety in size of SQB aggregates up to 40 µm, SQBs in Tm laser group were distinctly smaller, similar to SQBs in non-laser-treated skin. This is likely due to the different mechanisms of both lasers. The CO_2_ laser is ablative, creating micro-injuries in the skin that allow for deeper and more extensive penetration of HA. This enhanced penetration leads to the formation of larger aggregates. In contrast, the Tm laser operates without causing significant disruption to the corneal layer with the chosen settings, thereby limiting the amount of HA that can penetrate. This difference in skin interaction likely explains why the HA aggregate sizes observed with the Tm laser were smaller in comparison.

Our results suggest that both CO_2_ and Tm lasers significantly enhance HA uptake, with the CO_2_ laser achieving greater HA accumulation, potentially offering an advantage for improving skin quality. Fractional lasers, both ablative and non-ablative, are thought to induce beneficial healing responses by upregulating cytokines, increasing blood flow, and stimulating growth factors [14, 15]. Therefore, the therapeutic effect of laser-assisted HA delivery likely results from the combined impact of the wound healing response induced by the laser treatment and the effects of the SQB [16, 17].

This study employed clinically representative treatment settings. The CO_2_ laser induced a higher level of inflammation that persisted as a subclinical, inflamed metabolism even 30 days post-treatment. However, with subsequent SQB application, inflammation significantly decreased after 30 days, as observed through fluorescence lifetime microscopy. In contrast, skin treated with the Tm laser showed cellular metabolism levels similar to healthy, untreated control after 30 days, indicating a milder treatment to begin with. Thus, while the CO_2_ laser may be more effective in delivering HA and creating a more substantial inflamed environment with following skin remodeling, the Tm laser could be suitable for patients seeking minimal to no downtime.

The MPT technique is superior to conventional histology for assessing the intensity of intercellular edema due to the absence of artefact-inducing embedding, dehydration, and staining procedures [8, 18]. Furthermore, we were able to visualize and monitor HA distribution and morphologic and metabolic alterations in the long term in situ without the need for invasive or staining procedures [9]. By employing advanced MPT-FLIM techniques, we achieved high-resolution, non-invasive imaging that captures real-time dynamics of HA delivery and changes in human skin. This approach offers significant advantages over traditional methods, allowing for a comprehensive evaluation of both immediate effects over time of laser-assisted HA delivery.

This study has some limitations. Firstly, although we measured the size of HA aggregates, we did not quantify the amount of SQB delivered into the skin due to technical limitations of the MPT-FLIM. Additionally, we did not assess the potential systemic distribution of HA, which may differ between CO_2_ and Tm laser treatments. However, we used an HA product specifically intended for skin delivery, ensuring safety. The limited visualization depth of the MPT, potentially further restricted by inflammation-induced edema and laser scattering, may have missed alterations in the deeper dermal layers. Lastly, the age difference between the two intervention groups and the absence of topical anesthetic ointment in the Tm laser group may have influenced the results of the study.

In conclusion, this study not only underscores the efficacy of CO_2_ and Tm in enhancing HA uptake but also introduces a new approach for in vivo imaging and analysis of dermatological treatments. These insights pave the way for optimizing laser-assisted drug and cosmeceutical delivery systems, which will improve monitoring and therapeutic outcomes for various skin conditions and cosmetic applications. Further clinical studies are needed to investigate the comparative efficacy and safety of both treatment options.

## Data Availability

The data that support the findings of this study are available from the corresponding author upon reasonable request.

## References

[1] Dierickx C, Larsson MK, Blomster S (2018) Effectiveness and safety of acne scar treatment with nonanimal stabilized hyaluronic acid gel. Dermatol Surg 44:S10–S18 30358630 10.1097/DSS.0000000000001689

[2] Haedersdal M, Sakamoto FH, Farinelli WA, Doukas AG, Tam J, Anderson RR (2010) Fractional CO(2) laser-assisted drug delivery. Lasers Surg Med 42(2):113–122. 10.1002/lsm.20860 20166154 10.1002/lsm.20860

[3] Wenande E, Anderson RR, Haedersdal M (2020) Fundamentals of fractional laser-assisted drug delivery: An in-depth guide to experimental methodology and data interpretation. Adv Drug Deliv Rev 153:169–184. 10.1016/j.addr.2019.10.003 31628965 10.1016/j.addr.2019.10.003

[4] Nguyen L, Mess C, Schneider SW, Huck V, Herberger K (2023) In vivo characterization of laser-assisted delivery of hyaluronic acid using multiphoton fluorescence lifetime imaging. Exp Dermatol 32(12):2131–2137. 10.1111/exd.14961 37846872 10.1111/exd.14961

[5] Nisreen Mobayed B, Julie K, Jared JM (2020) Minimally invasive facial cosmetic procedures for the millennial aesthetic patient. J Drug Dermatol 19(1):100–103 10.36849/JDD.2020.4641 32395973

[6] Alexiades-Armenakas MR, Dover JS, Arndt KA (2008) The spectrum of laser skin resurfacing: nonablative, fractional, and ablative laser resurfacing. J Am Acad Dermatol 58(5):719–737 18423256 10.1016/j.jaad.2008.01.003

[7] Nguyen L, Mess C, Schneider SW, Huck V, Herberger K (2022) In vivo visualisation of tattoo particles using multiphoton tomography and fluorescence lifetime imaging. Exp Dermatol. 10.1111/exd.14646 35837813 10.1111/exd.14646

[8] König K (2008) Clinical multiphoton tomography. J Biophotonics 1(1):13–23 19343631 10.1002/jbio.200710022

[9] König K, Ehlers A, Stracke F, Riemann I (2006) In vivo drug screening in human skin using femtosecond laser multiphoton tomography. Skin pharmacology and physiology 19(2):78–88 16685146 10.1159/000091974

[10] Huck V, Gorzelanny C, Thomas K, Getova V, Niemeyer V, Zens K et al (2016) From morphology to biochemical state - intravital multiphoton fluorescence lifetime imaging of inflamed human skin. Sci Rep 6:22789. 10.1038/srep22789 27004454 10.1038/srep22789 PMC4804294

[11] Benzaquen M, Fongue J, Pauly V, Collet-Villette AM (2021) Laser-assisted hyaluronic acid delivery by fractional carbon dioxide laser in facial skin remodeling: a prospective randomized split-face study in France. Lasers Surg Med 53(9):1166–1172 33792961 10.1002/lsm.23403

[12] Mess C, Huck V (2018) Bedside assessment of multiphoton tomography: From skin cell morphology via fluorescence lifetime imaging to clinical pathophysiology. De Gruyter, Berlin, Germany. 10.1515/9783110429985-024

[13] Nino M, Calabro G, Santoianni P (2010) Topical delivery of active principles: the field of dermatological research. Dermatol Online J 16(1):4 20137746

[14] Laubach HJ, Tannous Z, Anderson RR, Manstein D (2006) Skin responses to fractional photothermolysis. Lasers in Surgery and Medicine: The Official Journal of the American Society for Laser Medicine and Surgery 38(2):142–149 10.1002/lsm.20254 16392146

[15] Orringer JS, Rittié L, Baker D, Voorhees JJ, Fisher G (2010) Molecular mechanisms of nonablative fractionated laser resurfacing. Br J Dermatol 163(4):757–768 20854401 10.1111/j.1365-2133.2010.09998.x

[16] Park SH, Kim DW, Jeong T (2012) Skin-tightening effect of fractional lasers: comparison of non-ablative and ablative fractional lasers in animal models. J Plast Reconstr Aesthet Surg 65(10):1305–1311. 10.1016/j.bjps.2012.04.028 22633871 10.1016/j.bjps.2012.04.028

[17] Russe E, Purschke M, Limpiangkanan W, Farinelli WA, Wang Y, Doukas AG et al (2018) Significant skin-tightening by closure of fractional ablative laser holes. Lasers Surg Med 50(1):64–69. 10.1002/lsm.22748 29058788 10.1002/lsm.22748

[18] Koenig K, Riemann I (2003) High-resolution multiphoton tomography of human skin with subcellular spatial resolution and picosecond time resolution. J Biomed Opt 8(3):432–439 12880349 10.1117/1.1577349

